# Electrohydrodynamic Drying of Chinese Wolfberry in a Multiple Needle-to-Plate Electrode System

**DOI:** 10.3390/foods8050152

**Published:** 2019-05-05

**Authors:** Jiabao Ni, Changjiang Ding, Yaming Zhang, Zhiqing Song, Xiuzhen Hu, Tingjie Hao

**Affiliations:** 1 College of Science, Inner Mongolia University of Technology, Hohhot 010051, China; nijiabao1227@163.com (J.N.); zym1610923951@163.com (Y.Z.); zqsong@imut.edu.cn (Z.S.); hxz@imut.edu.cn (X.H.); 2 Institute of Metrology and Testing of Inner Mongolia, Hohhot 010051, China; htj_wxh@163.com

**Keywords:** EHD drying, Chinese wolfberry, needle spacing, ionic wind

## Abstract

In order to systematically and comprehensively investigate electrohydrodynamic (EHD) drying characteristics and mechanisms in a multiple needle-to-plate electrode system, drying experiments of Chinese wolfberry were conducted by blocking ionic wind and changing needle spacing in a multiple needle-to-plate electrode system. Drying characteristics, quality parameters, and the microstructure of Chinese wolfberry fruits were measured. Results show that ionic wind plays a very important role during the drying process. Drying rates of different needle spacing treatments are significantly higher than that of the control, and the drying rate decreases with the increase of needle spacing. Needle spacing has a great influence on the speed of ionic wind, rehydration rate, and polysaccharide contents. The effective moisture diffusion coefficient and the electrical conductivity disintegration index decreases with an increase in needle spacing. Ionic wind has a great influence on the effective moisture diffusion coefficient and the electrical conductivity disintegration index of Chinese wolfberry fruits. The microstructure of Chinese wolfberry fruits dried in an EHD system significantly changed. This study provides a theoretical basis and practical guidance for understanding characteristic parameters and mechanisms of EHD drying technology.

## 1. Introduction

Chinese wolfberry fruit is widely consumed and has a range of medicinal properties [1,2,3,4,5]. Fresh fruits of Chinese wolfberry have a high moisture content and can easily rot or mildew; they are not suitable for long-term storage. So, it is very important to dry them in a timely manner. At present, the drying method of Chinese wolfberry mainly includes hot air drying [6], vacuum freeze drying [7], microwave drying [8], and solar drying [9]. Hot air drying and solar drying are easy to operate, but they easily destroy the effective nutrients in the wolfberry fruit and cause poor drying effect, poor color, and poor flavor quality. Microwave drying has a more rapid drying rate, but it can easily cause uneven drying of the materials. The effective nutrients and biological activities of Chinese wolfberry are maintained well using vacuum freeze drying, but the drying equipment is expensive, the operation is complicated, and the energy consumption is high. Therefore, it is imperative to explore the application of new drying technologies.

Electrohydrodynamic (EHD) drying technology is a new, nonthermal drying technology for food [10,11,12]. Esehaghbeygi and Basiry found that the EHD drying rate of tomato was twice as fast as that of air drying, and the surface temperature of the material was unchanged when a high voltage (10 kV) was applied [13]. Ding et al. investigated the drying characteristics of carrots in an EHD system and found that the drying rate of carrots was notably greater in the EHD system when compared to control at ambient temperature, and the drying quality was also improved when compared to oven drying at 70 °C [14]. Bai et al. found that EHD drying of sea cucumber at 45 kV resulted in better quality and consumed only 21.31% of the electric energy required for oven drying at 80 °C [15]. Chen et al. found that EHD drying had a very positive effect on inhibiting blueberry-degrading enzymes at a high voltage of 20 kV and a current around 0.16 mA [16]. Dinani et al. found that the rehydration capacity of mushroom slices with EHD treatment was significantly higher than that of the oven dried at 60 °C, but the shear strength was lower than that of the oven dried at 60 °C [17]. We found that the drying rate of the Chinese wolfberry with EHD treatment was notably greater than that of the control in previous research [18,19]. Compared with the wire-to-plate and plate-to-plate electrode systems, a needle-to-plate electrode configuration had the highest drying rate, and the active ingredient was preserved well [18,19]. Multiple mathematical models can be used to describe the drying characteristics of Chinese wolfberry with EHD treatment, but the best models are slightly different under different drying conditions [18,19]. Many experimental results in recent years indicated that the performance of EHD-enhanced drying was influenced by factors such as geometric characteristics, needle spacing, gap distance, applied voltage, and surface characteristics of materials under the drying process [20,21,22,23]. Yu et al. studied EHD drying of potato chips and found that potato chips were affected by different needle spacings and ionic winds [21]. Martynenko et al. studied the mechanism of EHD drying and found that, under the action of ionic winds, EHD drying could achieve the best drying effect when the needle spacing was 2–4 cm [22]. Zhang et al. studied the relationship between DC voltage and pin pitch in EHD drying and found that a four-pin pitch produced a higher ionic wind speed and had a higher drying rate than 12-pin and 28-pin pitches at low voltage. At high voltages, a 28-pin pitch produced a higher ionic wind speed and had a higher drying rate than four-pin and 12-pin pitches [23]. Although many researchers have investigated EHD drying technology, there are few systematic reports on the drying characteristics and mechanisms of Chinese wolfberry with multiple needle-to-plate electrodes in an EHD system. We think that, besides ionic wind, a nonuniform electric field also plays a role in the EHD drying process of Chinese wolfberry, but there is no experimental verification. So, it is necessary to conduct in-depth research.

In this paper, drying experiments of Chinese wolfberry were conducted through blocking ionic wind and changing needle spacing in a multiple needle-to-plate electrode system. The drying rate, water content, moisture diffusion coefficient, and the degree of cell damage during the drying process were measured. The effects of EHD drying on the polysaccharides and flavonoids in the dried products were studied. The microstructure of Chinese wolfberry during the drying process was investigated by means of scanning electron microscopy and infrared spectroscopy. This research provides experimental and theoretical bases for the application of EHD drying technology in the drying field of Chinese wolfberry.

## 2. Materials and Methods 

### 2.1. Experimental Equipment

**Figure 1:**
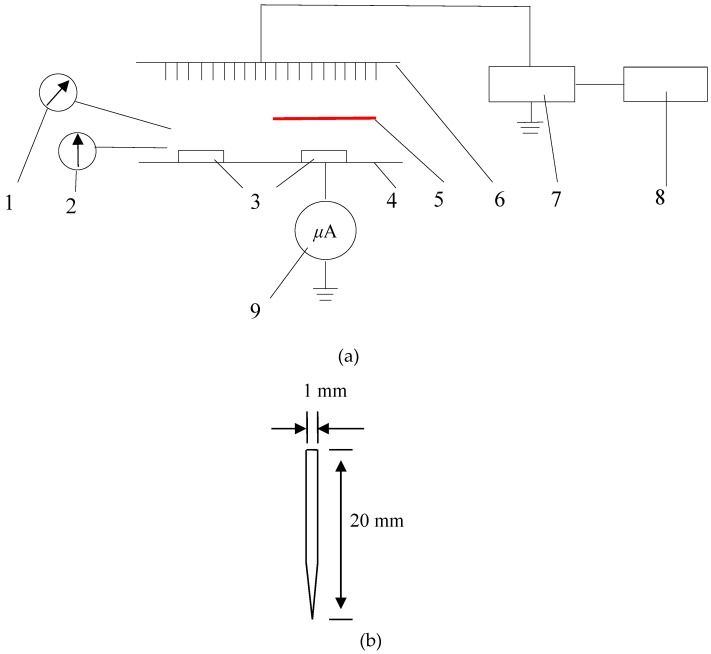
Schematic diagram of electrohydrodynamic (EHD) drying (**a**) and needle electrodes (**b**). 1. Hygrometer; 2. Thermometer; 3. Sample; 4. Ground electrode; 5. Insulating barrier medium; 6. Needle electrode; 7. High voltage power supply; 8. Control system; and 9. Microammeter.

The experimental equipment mainly consisted of a high-voltage power source, a voltage controller, and a multiple needle-to-plate electrode system. The high-voltage power source could output direct current (DC) voltage or alternating current (AC) voltage, and it was connected to a voltage controller with an adjustable voltage ranging from 0–70 kV for direct current (DC) or 0–50 kV for alternating current (AC). Multiple needle electrodes were connected to the power source. The distance between two needle electrodes could be adjusted. The needles were 20 mm long with a diameter of 1 mm. The ground electrode was a 100 cm × 45 cm stainless steel plate, and a microampere meter was connected between the ground electrode and the ground to measure the current generated during the drying experimental process. The distance between the needlepoint and the grounded electrode was 100 mm. Temperature and relative humidity were measured by a thermometer and a hygrometer, respectively, during the drying process. In order to detect drying effects caused by the ionic wind and the nonuniform electric field during the EHD drying process, we placed an insulating barrier between the multiple needle-to-plate electrode and the ground electrode to block ionic wind (Figure 1). 

### 2.2. Experimental Method

Fresh Chinese wolfberry fruits were purchased from growers in the county of Tuoketuo, Hohhot, Inner Mongolia, China, and they were immediately placed in a refrigerator maintained at 4 °C. Refrigerated Chinese wolfberry was immersed in a 5% sodium carbonate solution at a temperature of 50 °C. After 10 min, the fresh Chinese wolfberry were taken out of the solution. Excess water of the Chinese wolfberry was completely removed with absorbent paper. Then, we determined the initial moisture content of the pretreated Chinese wolfberry with a rapid moisture analyzer (Shanghai Sh10A) and investigated the EHD drying experiment.

The initial moisture content of the pretreated Chinese wolfberry was 76% ± 1%. 

The EHD drying conditions were as follows:

Firstly, to detect drying effects caused by the ionic wind and the nonuniform electric field during the EHD drying process, the pretreated Chinese wolfberry was divided into three parts to conduct the drying experiments: the first part was directly placed in an EHD system, the second part was placed below the insulating barrier in the same EHD system, and the last part was placed in the environment and taken as the control. The corresponding voltage, electrode distance, and needle spacing were 28 kV, 100 mm, and 40 mm, respectively. The drying temperature, relative humidity, and ambient wind speed were 21 ± 2 °C, 30% ± 5%, and 0 m/s, respectively.

Secondly, to detect drying effects caused by needle spacing during the EHD drying process, the needle spacing was changed each time to 2, 4, 6, 8, 10, and 12 cm. The corresponding voltage and electrode distance were 28 kV and 100 mm, respectively. 

In the above experiment, the mass of Chinese wolfberry in the drying process was measured by electronic balance (BS124S, Sartorius Scientific Instrument Co., Ltd., Beijing, China) every hour. The moisture ratio and drying rate were calculated using the corresponding formulas. Each experiment was repeated three times independently, and the results were expressed as mean ± standard deviation (SD).

### 2.3. Measurement of Related Parameters

#### 2.3.1. Ion Wind Speed

The ionic wind speed under different experimental conditions was measured using a thermal anemometer probe (405i, Ruice Electronics Technology Co., Ltd., Guangzhou, China). Each experiment was repeated three times independently, and the average was taken.

#### 2.3.2. Moisture Ratio

The moisture content and moisture ratio of the Chinese wolfberry fruit during the drying process are defined as:

(1) $$M_{i}=\frac{m_{i}-m_{g}}{m_{g}}\times 100\%,$$

(2) $$\mathrm{MR}=\frac{M_{i}-M_{e}}{M_{0}-M_{e}},$$

where $m_{g}$ is the dry mass of Chinese wolfberry, $m_{i}$ is the mass of the Chinese wolfberry fruit at time ti, $M_{0}$ is the water content of the Chinese wolfberry fruit at time t_0_, $M_{i}$ is the water content of the Chinese wolfberry fruit (g water/g dry matter) at time ti, $M_{e}$ is the equilibrium water content of the Chinese wolfberry fruit, and MR is the moisture ratio of the Chinese wolfberry fruit.

#### 2.3.3. Drying Rate

The drying rate of Chinese wolfberry fruit in the drying process is defined as:

(3) $$\mathrm{DR}=\frac{M_{t}-M_{t\;+\;\Delta t}}{\Delta t},$$

where DR is the drying rate (g water/g dry matter × h), $M_{t}$ is the water content of the Chinese wolfberry fruit at time t, and $M_{t\;+\;\Delta t}$ is the water ratio of the Chinese wolfberry fruit at time t+Δt.

#### 2.3.4. Shrinkage Rate

The shrinkage rate was measured by displacement using distilled water as a control solution. Distilled water (50 mL) was put into the measuring cylinder. Then, Chinese wolfberry fruit or dried fruit product was placed into the measuring cylinder, and the Chinese wolfberry was completely immersed in distilled water. The total volume of the Chinese wolfberry fruit and distilled water was measured. The difference between the total volume and the volume of distilled water was the volume of Chinese wolfberry fruit. 

The shrinkage rate of Chinese wolfberry is calculated using the following equation:

(4) $$\mathrm{SR}=\frac{V_{0}-V_{f}}{V_{0}}\times 100\%,$$

where SR is the shrinkage rate of the Chinese wolfberry fruit, $V_{0}$ is the volume of the fresh Chinese wolfberry fruit, and $V_{f}$ is the volume when the Chinese wolfberry fruit moisture content reaches 10%.

#### 2.3.5. Rehydration Rate

Ten grams of dried Chinese wolfberry was immersed in 100 mL distilled water at 37 °C in a water bath for 7 h. Then, the Chinese wolfberry fruit was removed, and the surface water was completely absorbed with filter paper. The Chinese wolfberry fruits were weighted by electronic balance after removing excess water with absorbent paper. The rehydration rate of Chinese wolfberry fruit is calculated using the following equation:

(5) $$\mathrm{RR}=\frac{m_{a}}{m_{b}},$$

where RR is the rehydration rate of Chinese wolfberry fruit, $m_{a}$ is the mass of the dried Chinese wolfberry fruits after rehydration, and $m_{b}$ is the mass of the dried Chinese wolfberry fruits before rehydration.

#### 2.3.6. Electrical Conductivity Disintegration Index

The electrical conductivity of Chinese wolfberry fruit was measured immediately after EHD treatment using a conductivity meter (SIN-CT-TDS3031, Sinomeasure Automation Technology Co., Ltd., Hangzhou, China). The degree of cell damage, expressed as the electrical conductivity disintegration index, Z*p*, was estimated on the basis of electrical conductivity values. Z*p* is calculated according to the following equation:

(6) $$Z_{P}=\left(\sigma -\sigma_{i}\right)/\left(\sigma_{d}-\sigma_{i}\right),$$

where *σ* is the conductivity of the Chinese wolfberry in different conditions, *σ_i_* is the conductivity of nontreated (intact) tissue by electric field, and *σ_d_* is the conductivity of Chinese wolfberry corresponding to completely destroyed sample treated by electric field. 

#### 2.3.7. Effective Moisture Diffusion Coefficient

The effective moisture diffusion coefficient of Chinese wolfberry during the drying process was calculated using Fick’s second law. The equation is:

(7) $$\frac{dM}{dt}=D_{eff}\frac{d^{2}M}{dr^{2}}.$$

For a long drying process, MR < 0.6, the equation can be expressed as:

(8) $$\mathrm{MR}=\frac{8}{\pi^{2}}exp(-\frac{\pi^{2}D_{eff}t}{4L^{2}}),$$

where *D_eff_* is the effective diffusion coefficient of the Chinese wolfberry and *L* is the thickness of Chinese wolfberry.

#### 2.3.8. Infrared Spectrum

The dried sample was ground into fine powder in an agate mortar. The sample powder was uniformly mixed with potassium bromide at a 1:100 ratio and filtered with 80 target quasi-sieves. It was pressed into disks using a tablet machine (HY-12, Tianguang Spectrometer Co., Ltd., Tianjin, China). The sample was scanned using a Fourier transform infrared spectrometer (Nicolet iS10, Thermo Nicolet Corporation, New York, NY, USA). 

#### 2.3.9. Scanning Electron Microscopy

To investigate the interior structure of Chinese wolfberry fruits with ion wind and nonuniform electric field treatments, a small piece of sample was cut, kept on an aluminum base, and photographed using a scanning electron microscope (S3400, Hitachi corporation, Tokyo, Japan).

#### 2.3.10. Flavonoid Content

Total flavonoid content was determined with a spectrophotometric method [24,25]. The dried Chinese wolfberry fruits were ground into fine powder. A 1.0 g sample was placed into a 150 mL conical flask with a stopper. Then, 30 mL of methanol solution was added. The conical flask was shook for 2 h at 160 r/min and 65 °C in a thermostatic water bath oscillator. Then, the mixed liquor was filtered, and the filtered liquor was transferred into a 50 mL volumetric flask. Then, we added methanol solution to obtain a total volume of 50 mL. We used the standard rutin solution to set the standard curve. The absorbance of this solution was measured at 420 nm according to the steps of the standard curve. 

#### 2.3.11. Polysaccharide Content

Ultrasound-assisted extraction of polysaccharides was conducted following the method proposed by Yang et al. [18]. Ultrasonic extraction technology is an extraction technique that uses mechanical disruption and cavitation of ultrasonic waves. This dissolved the Chinese wolfberry fruit cell wall and accelerated the diffusion of the extract from the raw material to the solvent. When an ultrasonic wave that reaches a certain sound intensity propagates in a liquid, the liquid medium is continuously stretched and compressed to form cavitation bubbles. Cavitation bubbles continue to increase during the stretching and compression process, and they collapse to about twice their initial volume, instantaneously producing multiple effects such as strong cavitation, high shear, crushing, and agitation. It promotes swelling and solvation of the plant material, resulting in an increase in the pore size of the cell wall, which promotes release of internal products such as cell polysaccharides. In addition, the free radical effect can break hydrogen bonds, destroy cross-linking between molecules, and destroy the cell wall, which improves the polysaccharide extraction rate and shortens extraction time.

Dried Chinese wolfberry fruits were ground into a fine powder. One gram of Chinese wolfberry was placed into a centrifuge tube. Then, 5 mL of distilled water and 20 mL of absolute ethyl alcohol were added into the centrifuge tube. Samples underwent sonication for 30 min using an ultrasonic processor. After sonication, the extract was centrifuged at 4000 rpm for 10 min. We separated the residue and supernatant, and we collected the precipitate. Ten milliliters of ethanol was then added, and the precipitate was washed and centrifuged. We transferred the precipitate into a round-bottom flask and added 50 mL of distilled water. The samples were extracted in a boiling water bath. After 2 h, the supernatant for the samples was transferred into a 100 mL volumetric flask and kept at a constant volume with distilled water. We used a standard glucose solution to set the standard curve. The absorbency of this solution was measured at 490 nm according to the steps of the standard curve.

#### 2.3.12. Statistical Analysis

One-way ANOVA was used to calculate the moisture ratio and drying rate between the Chinese wolfberry fruits under different EHD drying conditions and the control. The statistical significance employed was *p* < 0.05. The effective moisture diffusion coefficient, polysaccharide content, and flavonoid content were also calculated between the Chinese wolfberry fruits treated under different EHD drying conditions and the control using one-way ANOVA. The results reported in this study were presented as mean ± standard deviation (SD).

## 3. Results

### 3.1. Measurement Results of Ionic Wind Speed 

**Figure 2:**
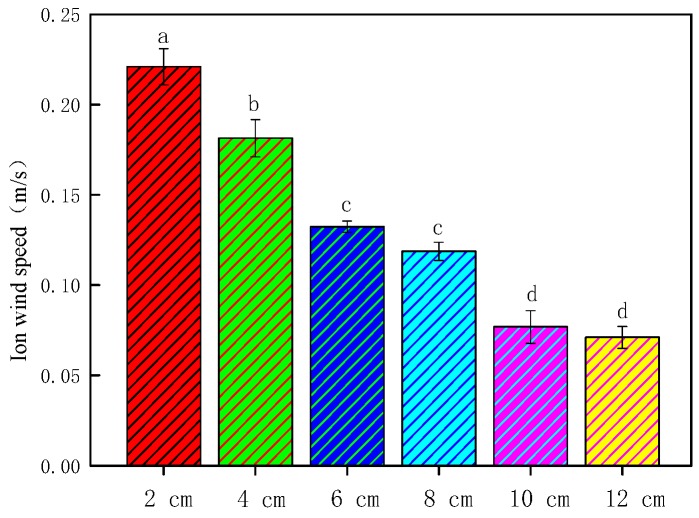
Variation in ionic wind speeds under different needle spacings. Data are shown as the mean ± standard deviation (SD). For each response, means with different lower-case letters are significantly different (*p* < 0.05).

Ionic wind speed was greatly reduced after being blocked by the insulating medium; it changed from 0.1814 to 0.0110 m/s. So, ionic wind could be neglected after being blocked. Figure 2 depicts the measurement results of ionic wind speeds under different needle spacings. As shown in Figure 2, the ionic wind speeds were 0.2210, 0.1814, 0.1323, 0.1186, 0.0768, and 0.0710 m/s, respectively, at 2, 4, 6, 8, 10, and 12 cm needle spacings. Ionic wind speed decreased with the increase in needle spacing. 

### 3.2. Effect of Electrohydrodynamic (EHD) Drying on the Moisture Ratio of Chinese Wolfberry Fruits

**Figure 3:**
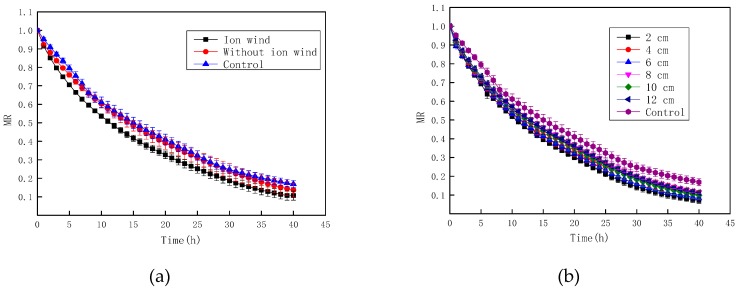
The moisture ratio of Chinese wolfberry fruits. (**a**) Ionic wind and without ionic wind; (**b**) different needle spacing.

Figure 3 depicts the moisture ratio of Chinese wolfberry fruits during the drying process. It was seen in Figure 3a that the decline of moisture ratio with ionic wind treatment was the fastest, and the control group was the slowest. By ANOVA, the results showed that the moisture ratio with ionic wind treatment was significantly different from that without ionic wind treatment and the control (*p* < 0.05). However, there was no significant difference between nonionic wind treatment and the control (*p* > 0.05). As seen from Figure 3b, the decline of the moisture ratio was significantly faster under EHD compared with that of the control, and the decline rate decreased with the increase of needle spacing. The traditional drying methods for Chinese wolfberry fruits are mainly sun drying and hot air drying. The drying time for sun drying is more than 96 h, and the drying time for hot air drying is about 48 h. It was also seen from Figure 3 that the drying time in an EHD system was about 40 h when the moisture content of Chinese wolfberry fruits reached 10%. So, for the drying time, it satisfied the industrial demand. If EHD drying technology were to be combined with other drying technologies, the Chinese wolfberry drying time would be shortened. However, this requires further study.

### 3.3. Effect of EHD Drying on the Drying Rate of Chinese Wolfberry Fruits

**Figure 4:**
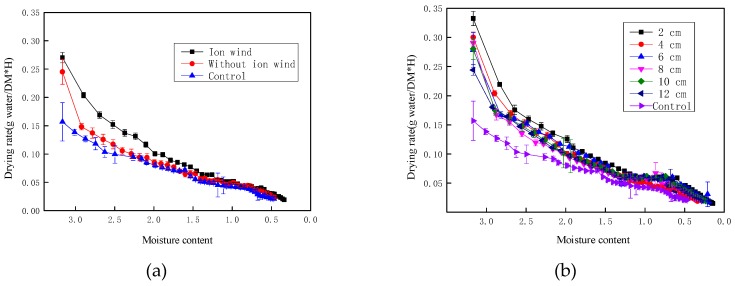
The drying rate of Chinese wolfberry fruits. (**a**) Ionic wind and without ionic wind; (**b**) different needle spacing.

Figure 4 depicts the drying rate of Chinese wolfberry fruits during the drying process. It was seen from Figure 4a that the drying rate of Chinese wolfberry with ionic wind treatment was the highest, and the control group was the lowest. By ANOVA, the results showed that the drying rate with ionic wind treatment was significantly different from that without ionic wind treatment and the control (*p* < 0.05). However, there was no significant difference between nonionic wind treatment and the control (*p* > 0.05). The surface moisture of the Chinese wolfberry fruits was continuously blown into the external environment by ionic wind. Then, the moisture gradient of the Chinese wolfberry fruits increased. The nonuniform electric field destroyed the cell membrane and changed the permeability of Chinese wolfberry fruits. So, the drying speed of the material was significantly improved. From Figure 4b, we saw that the drying rate of Chinese wolfberry fruits with EHD treatment was significantly faster than that of the control. The drying rates of Chinese wolfberry fruits exposed to EHD treatments at six levels of needle spacing were improved by factors of 1.4676, 1.3688, 1.3264, 1.3085, 1.3039, 1.278, and 1.2367, respectively, at 2, 4, 6, 8, 10, and 12 cm, compared to that of the control, in the first 10 h. The drying rate of Chinese wolfberry fruits decreased with the increase of needle spacing. From Figure 3 and Figure 4, we saw that as the moisture ratio of dry products decreased, the drying rate of the Chinese wolfberry also decreased. 

### 3.4. Effect of EHD Drying on the Rehydration Rate

**Figure 5:**
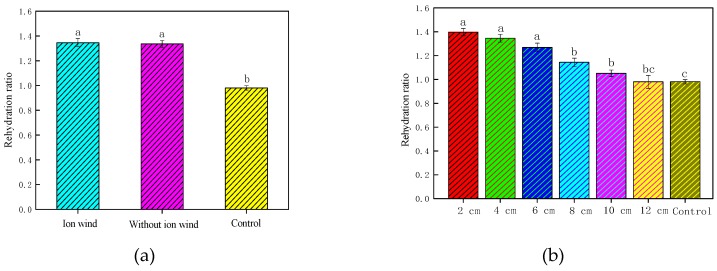
The rehydration rate of Chinese wolfberry fruits. (**a**) Ionic wind and without ionic wind; (**b**) different needle spacing. Data are shown as the mean ± SD. For each response, means with different lower-case letters are significantly different (*p* < 0.05).

Figure 5 depicts the rehydration rate of Chinese wolfberry fruits under different experimental conditions. It was seen from Figure 5a that the rehydration rate with EHD treatment was higher than that of the control. It also indicated that the nonuniform electric field had a significant effect on the rehydration rate of the Chinese wolfberry fruits during the EHD drying process, but the ionic wind had little effect on the rehydration rate of the Chinese wolfberry fruits. From Figure 5b we saw that the rehydration rates under different needle spacings were higher than that of the control. As the needle spacing increased, the rehydration rate gradually decreased.

### 3.5. Effect of EHD Drying on the Shrinkage Rate

**Figure 6:**
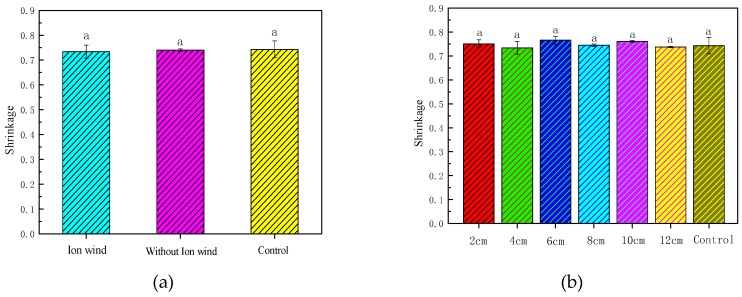
The shrinkage rate of Chinese wolfberry fruits. (**a**) Ionic wind and without ionic wind; (**b**) different needle spacing. Data are shown as the mean ± SD. For each response, means with different lower-case letters are significantly different (*p* < 0.05).

Figure 6 depicts the shrinkage rate of Chinese wolfberry fruits under different experimental conditions. The shrinkage rates of Chinese wolfberry fruits were 0.73367, 0.73976, and 0.7432, respectively, for the ionic wind treatment, without ionic wind treatment, and the control. The shrinkage rates of Chinese wolfberry fruits were 0.75057, 0.73367, 0.76567, 0.7446, 0.7602, and 0.73697, respectively, at 2, 4, 6, 8, 10, and 12 cm. This showed that there was no significant difference of the shrinkage rates of Chinese wolfberry fruits with EHD treatment.

### 3.6. Polysaccharide Content of Chinese Wolfberry Fruits

Polysaccharides are one of the effective nutrients of Chinese wolfberry. They have important functions, such as regulating immunity, and important anti-aging, anti-tumor, and anti-oxidation properties [26,27]. Therefore, preservation of polysaccharides is an important indicator when evaluating the drying technique. Table 1 and Table 2 show the polysaccharides content of Chinese wolfberry fruits under different conditions. It was seen from Table 1 and Table 2 that all EHD drying treatment groups did not destroy the polysaccharide content of Chinese wolfberry, and ionic wind had no significant effect on the polysaccharide content. When needle spacing was 4 cm, polysaccharide content was the highest. Polysaccharide content was lowest at 10 cm and 12 cm. It was seen from the experimental results that polysaccharide content gradually decreased with the increase of needle spacing. We thought that there was a negative correlation between polysaccharide content and drying time of Chinese wolfberry. As the needle spacing increased, the total drying time increased. Then, the polysaccharide content decreased. Table 1 and Table 2 indicated that the polysaccharide content of Chinese wolfberry was largely preserved in the nonuniform electric field. 

### 3.7. Flavonoid Content of Chinese Wolfberry Fruits

Flavonoids are one of the most important active ingredients in Chinese wolfberry fruits. They have a variety of biological benefits such as antioxidation, antiaging, and anticancer as well as biological functions such as lowering blood sugar, improving human immunity, etc. [28,29,30,31]. Table 3 and Table 4 show flavonoid contents of Chinese wolfberry fruits under different conditions. It was seen from Table 3 and Table 4 that all EHD treatments had no significant difference on flavonoid contents of Chinese wolfberry fruit, and ionic wind had no significant effect on flavonoid contents. Song et al. studied freeze-dried and hot air-dried goji and found that, compared with fresh goji, freeze drying and hot air drying significantly increased goji flavonoid content [32]. Our previous research found that there was no significant difference in flavonoid content between EHD drying and the control [18]. Flavonoids are mainly composed of naringenin chalcone, flavanol rutin, and a quercetin glycoside. The enzyme responsible for oxidation of phenolic substances is inactivated at high temperatures [33]. Moreover, phenolic compounds in fruits are more concentrated outside the cell rather than the vacuoles. At high temperatures, liberation of phenolics might occur as a result of the decomposed cellular components and covalent bonds [34,35]. However, EHD drying did not cause temperature changes, so there was no significant difference from the control group.

### 3.8. The Electrical Conductivity Disintegration Index of Chinese Wolfberry Fruits

**Figure 7:**
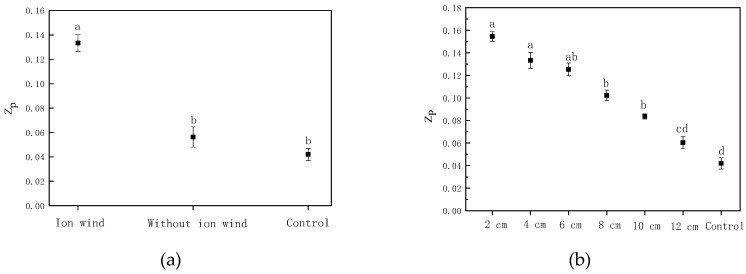
The electrical conductivity disintegration index of Chinese wolfberry fruits. (**a**) Ionic wind and without ionic wind; (**b**) different needle spacings. Data are shown as the mean ± SD. For each response, means with different lower-case letters are significantly different (*p* ≤ 0.05).

Under EHD drying, the maximum membrane voltage was quickly reached on the cell membrane, and the cell membrane was thinned by the force of the electric field. When it reached a certain critical value, it was broken down, causing the cell membrane to disintegrate and produce fine voids, resulting in extracellular components. Entry of substances caused the cells to rupture, which in turn changed the permeability of the cell membrane and accelerated evaporation of water inside the cells. The higher the voltage, the larger the pores of the cell membrane were, and the better the permeability was, which affected the drying speed. The electrical conductivity disintegration index was determined by the degree of damage caused by EHD drying on tissue. Figure 7 depicts the electrical conductivity disintegration index of Chinese wolfberry fruits under different conditions. It was seen from Figure 7 that the electrical conductivity disintegration index of Chinese wolfberry fruits subjected to ionic wind was greatly higher than that of nonionic wind and the control. The electrical conductivity disintegration index of Chinese wolfberry fruits in different needle spacings was also significantly higher than that of the control, and it gradually decreased as the needle spacing increased. This indicated that ionic wind had greater influence on the electrical conductivity disintegration index compared with the nonuniform electric field. According to previous research, ionic wind speed was correlated with needle spacing, which also directly affected the electrical conductivity disintegration index of Chinese wolfberry fruits. Increase of the electrical conductivity disintegration index was beneficial to the diffusion of water molecules in the material. So, this increased the drying rate of Chinese wolfberry.

### 3.9. The Effective Moisture Diffusion Coefficient of Chinese Wolfberry Fruits

Table 5 and Table 6 depict the effective moisture diffusion coefficient of Chinese wolfberry fruits under different conditions. It was seen from Table 5 and Table 6that the effective moisture diffusion coefficient inside the Chinese wolfberry fruit was the highest with ionic wind. The effective moisture diffusion coefficient of Chinese wolfberry fruits in different needle spacings was higher than that of the control, and it decreased with the increase of needle spacing. Martynenko et al. studied EHD drying of grape pomace and found that the average effective moisture diffusion coefficient with the EHD treatment was higher than that of convective drying [36]. Dinani et al. found that the average effective internal moisture diffusion coefficient of mushrooms with EHD treatment was higher than that of the control, and the average internal moisture effective diffusion coefficient decreased as the needle spacing increased, which was similar to our experimental results [37].

Water molecules inside the cells of Chinese wolfberry fruits are a polar molecule, and water molecules are mainly hydrogen-bonded to form a molecular group, When Chinese wolfberry fruit was dried in a nonuniform electric field, the water molecules of the cells were subjected to a nonzero force. This caused an increased acceleration of the water molecules, an increased speed of movement, a continuous increase in the energy carried, and hydrogen bonds between water molecules were promoted to be broken. Water molecules in the cell overcame various resistances to gradually permeate the surface of the Chinese wolfberry fruit, and then the action of the ion wind continuously blew off the water molecules on the surface of the Chinese wolfberry fruit to the external environment, thereby accelerating the drying effect.

### 3.10. Analysis of Infrared Spectrum

**Figure 8:**
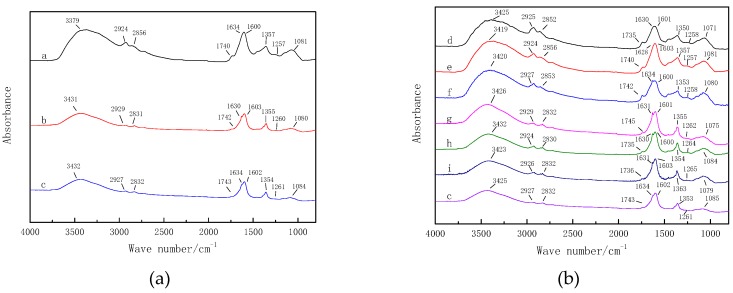
Infrared spectra of Chinese wolfberry fruits under ion wind and without ion wind. (**a**) Ionic wind and without ionic wind; (**b**) different needle spacing. Note: a. Ionic wind; b. Without ionic wind; c. Control; d. 2 cm; e. 4 cm; f. 6 cm; g. 8 cm; h. 10 cm; and i. 12 cm.

Figure 8 depicts infrared spectrum of Chinese wolfberry fruits under different conditions. Table 4 depicts classification of the main absorption characteristic peaks of the infrared spectrum of Chinese wolfberry fruits. It can be seen from Figure 8a that the infrared spectrum of Chinese wolfberry fruits with the ionic wind and the nonuniform electric field treatments had the same peak position and the same chemical composition, but the peak intensity of the ionic wind treatment was higher than that of the nonuniform electric field treatment and the control. However, the position and intensity of the spectrum peaks were approximately the same as the nonuniform electric field treatment and the control. This indicated that ionic wind had great influence on the intensity of the characteristic absorption peak of the infrared spectrum. From a microscopic point of view, compared with the nonuniform electric field, the ion wind effect had a greater influence on the dipole moment and vibration mode, which affected the preservation of nutrients. In summary, ion wind had a greater influence on the characteristic peak intensity of the infrared spectrum of Chinese wolfberry fruits. It was seen from Figure 8b that the infrared spectral characteristic peaks of the different needle spacings were substantially similar, and the characteristic peak intensity decreased as the needle spacing increased. The characteristic peak intensity was higher compared to the control. The chemical composition of Chinese wolfberry fruits in different needle spacings and the control was basically the same, but the nutrient contents were preserved better than that of the control. From Table 7, it was found that the stretching vibrations of N-H and O-H of polysaccharides, glycosides, amino acids, proteins, and sugar alcohols were in the vicinity of 3420 cm^−1^; the wave numbers at 2927 cm^−1^ and 2855 cm^−1^ were the telescopic vibrations of the C-H of methylene and methyl, respectively; the wave number at 1740 cm^−1^ was the telescopic vibration of C=O of carboxylic acids or esters; the wave numbers at 1630, 1380, and 1250 cm^−1^ were the vibrations of amino acids, the protein amide I band, III band, alkaloids, and unsaturated esters; and the broad, strong peak near 1060 cm^−1^ was mostly the bending vibration of C-OH of carbohydrates, such as glycosides and polysaccharides. According to Figure 8 and Table 4, we saw that the internal active components of Chinese wolfberry fruits were affected using EHD drying. This is of great help to further the study of the Electrohydrodynamic (EHD) drying mechanism.

### 3.11. The Surface Microstructure of Chinese Wolfberry Fruits

**Figure 9:**
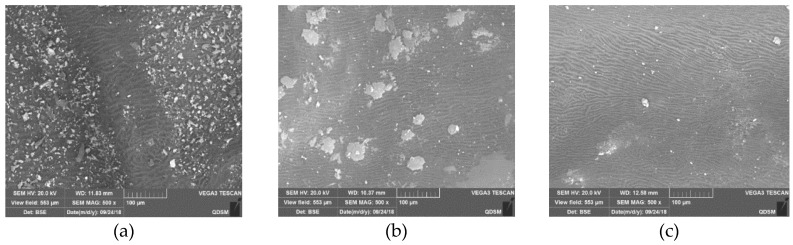
Surface microstructures of Chinese wolfberry fruits. (**a**) Ionic wind; (**b**) without ionic wind; (**c**) control.

Figure 9 depicts the surface microstructure of Chinese wolfberry fruits. Results showed that there were significant differences under different drying conditions. The surface of Chinese wolfberry fruits had a large number of very small crystals under ionic wind treatment. Without ionic wind, a number of large crystals appeared on the surface. And the control was relatively regular with only a very small number of crystals appearing on the surface. This indicated that the nonuniform electric field and ionic wind using EHD drying technology greatly influenced the surface microstructure of Chinese wolfberry fruits.

## 4. Discussion

The average drying rate of the Chinese wolfberry with ionic wind treatment was 0.072 g/(g × h), and the average drying rate of the Chinese wolfberry without ionic wind treatment was only 0.067 g/(g × h). The electrical conductivity disintegration index of the Chinese wolfberry with ionic wind treatment was 0.133, and the electrical conductivity disintegration index of the Chinese wolfberry without ionic wind treatment was 0.056. The electrical conductivity disintegration index of the Chinese wolfberry with ionic wind treatment was 2.375 times that of the Chinese wolfberry without ionic wind treatment. The effective moisture diffusion coefficient of the Chinese wolfberry with ionic wind treatment was 4.621087 × 10^−10^ m^2^/s, and the effective moisture diffusion coefficient of the Chinese wolfberry without ionic wind treatment was only 3.737270 × 10^−10^ m^2^/s. From the above research, it was found that ionic wind had a greater influence on characteristic parameters under the drying process, such as drying rate, moisture ratio, the electrical conductivity disintegration index, the effective moisture diffusion coefficient, etc. The rehydration rate, shrinkage rate, polysaccharide content, and flavonoid content of the Chinese wolfberry without ionic wind treatment were 1.334, 0.739, 15.3 (g/100 g), and 0.10 (g/100 g), respectively. However, the rehydration rate, shrinkage rate, polysaccharide content, and flavonoid content of the Chinese wolfberry with ionic wind treatment were 1.345, 0.733, 16.0 (g/100 g) and 0.10 (g/100 g), respectively. From the above experimental results, ionic wind had no significant impact on these parameters, and more impact may come from the nonuniform electric field. So, the nonuniform electric field has a greater influence on the quality parameters under the drying process, such as rehydration rate, shrinkage rate, polysaccharide content, flavonoid contents, etc. Ionic wind had a greater influence on the infrared spectrum characteristic absorption peak intensity and the surface microstructure of Chinese wolfberry fruits. Furthermore, both ionic wind and the nonuniform electric field play a very important role and play different roles under the process of EHD drying. Ionic wind greatly increased the drying rate of Chinese wolfberry fruits, while the nonuniform electric field greatly preserved the loss of effective components. Many researchers have suggested that the mechanism of EHD drying was mainly the action of ionic wind [22,38,39], which was consistent with our experimental results. But they ignored the effect of nonuniform electric fields. We think that the mechanism of EHD drying is the interaction of ionic wind and a nonuniform electric field.

For a single needle-to-plate electrode, the distribution of ions can be calculated using the following equation [22]:

(9) $$j\left(\theta \right)=j\left(0\right){cos}^{m}\theta$$

**Figure 10:**
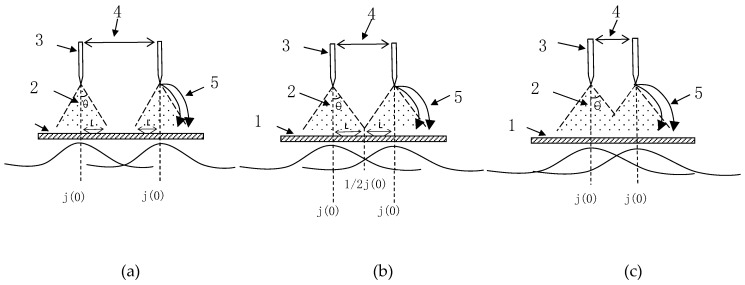
Current density distribution of ions. (**a**) without interference; (**b**) exact distance; (**c**) with interference.1. Ground electrode; 2. electrode distance; 3. emitter electrode; 4. distance between two neighbored electrodes; and 5. field and ionic flow line.

For multiple needle-to-plate electrodes, the distribution of ions is superimposed by multiple single needle electrodes. The distribution figures (Figure 10) are as follows:

The difference in needle spacing changes the average ionic wind speed. As needle spacing decreases, the ionic wind speed increases. Ionic wind speed ranged approximately from 0.07 to 2.2 m/s under different needle spacings. From the above research, we found that needle spacing had a greater influence on the drying characteristic parameters, the electrical conductivity disintegration index, the effective moisture diffusion coefficient, the and infrared spectrum characteristic absorption peak intensity. For example, as the needle spacing increases, the ion wind speed, the electrical conductivity disintegration index, the effective moisture diffusion coefficient, and the infrared spectrum characteristic absorption peak intensity are gradually reduced. This result also showed that enhancement of the mass transfer rate could be attributed to corona wind. Enhancement of the drying rate by the multiple point-to-plate electrode could be attributed to electric wind created by each needle point electrode, resulting in a cumulative effect that could have greatly increased the drying rate [11,38], which was consistent with our experimental results.

## 5. Conclusions

The drying rate and the dried quality of Chinese wolfberry fruits under EHD treatment were significantly higher than that of the control. Ionic wind had a greater influence on the drying characteristic parameters of Chinese wolfberry fruits, and the nonuniform electric field had a greater influence on the quality parameters under the drying process. With the increase of needle spacing, the drying rate of Chinese wolfberry fruits gradually decreased. Taken together, it can be concluded that the mechanism of EHD drying is the interaction of ionic wind and a nonuniform electric field.

## Figures and Tables

**Table 1:** Polysaccharide content under ionic wind and without ionic wind (g/100 g).

	Ionic Wind	Without Ionic Wind
Polysaccharides	16.0 ± 0.03 ^a^	15.3 ± 0.05 ^a^

Note: Data are shown as the mean ± standard deviation (SD). For each response in this table, means in each column that do not share the same lowercase letters are significantly different (*p* < 0.05).

**Table 2:** Polysaccharide content (g/100 g) under different needle spacings.

Needle Spacing	Control	2 cm	4 cm	6 cm	8 cm	10 cm	12 cm
Polysaccharides	12.9 ± 0.03 ^a^	12.6 ± 0.06 ^a^	16.0 ± 0.03 ^b^	15.3 ± 0.01 ^b^	13.7 ± 0.04 ^b^	12.3 ± 0.02 ^a^	12.3 ± 0.04 ^a^

Note: Data are shown as the mean ± standard deviation (SD). For each response in this table, means in each column that do not share the same lowercase letters are significantly different (*p* < 0.05).

**Table 3:** Flavonoid content under ionic wind and without ionic wind (g/100 g).

	Ion Wind	Without Ion Wind
Flavonoids	0.10 ± 0.008 ^a^	0.10 ± 0.006 ^a^

Note: Data are shown as the mean ± SD. For each response in this table, means in each column that do not share the same lowercase letters are significantly different (*p* < 0.05).

**Table 4:** Flavonoid content (g/100 g) under different needle spacings.

Needle Spacing	Control	2 cm	4 cm	6 cm	8 cm	10 cm	12 cm
Flavonoids	0.11 ± 0.006 ^a^	0.13 ± 0.003 ^a^	0.10 ± 0.008 ^a^	0.08 ± 0.004 ^a^	0.12 ± 0.004 ^a^	0.09 ± 0.008 ^a^	0.12 ± 0.005 ^a^

Note: Data are shown as the mean ± SD. For each response in this table, means in each column that do not share the same lowercase letters are significantly different (*p* < 0.05).

**Table 5:** Effective moisture diffusion coefficients under ionic wind and without ionic wind.

	Ionic Wind	Without Ionic Wind	Control
*D_eff_* (10^−10^ m^2^/s)	4.621087 ± 0.000015 ^a^	3.737270 ± 0.000052 ^b^	3.655958 ± 0.000121 ^b^

Note: Data are shown as the mean ± SD. For each response in this table, means in each column that do not share the same lowercase letters are significantly different (*p* < 0.05).

**Table 6:** Effective moisture diffusion coefficients under different needle spacings.

Needle Spacing (cm)	*D_eff_* (10^−10^ m^2^/s)
2	4.740000 ± 0.000029 ^a^
4	4.621087 ± 0.000015 ^a^
6	4.477926 ± 0.000018 ^a^
8	4.148498 ± 0.000041 ^b^
10	4.032420 ± 0.000014 ^b^
12	3.943630 ± 0.000025 ^b^
Control	3.655958 ± 0.000121 ^c^

Note: Data are shown as the mean ± SD. For each response in this table, means in each column that do not share the same lowercase letters are significantly different (*p* < 0.05).

**Table 7:** Classification of the main absorption characteristic peaks of the infrared spectrum of Chinese wolfberry fruits.

Wave Number (cm^−1^)		Classification
3420	ν(O-H)	Polysaccharides, Glycosides, and Sugar alcohols
	ν(N-H)	Amino acids and Proteins
3010	ν(=CH)	Fatty acids and Alkanes
2960, 2870	Methylν(C-H)	
2927, 2855	Methyleneν(C-H)	
1740	ν(C=O)	Carboxylic acids and esters
1630, 1380, 1250	ν(C=C)	Amino acid, Protein amide I band, III band,
	*ν*(C=O-C)	Alkaloids, and Unsaturated ketones
1060	*δ*(C-OH)	Carbohydrates such as glycosides and polysaccharides

ν: Telescopic vibration or skeleton vibration. δ: Bending vibration.
